# Supplementary material for: A fast impedance-based antimicrobial susceptibility test
Source: Nat Commun. 2020 Oct 21;11:5328. doi: 10.1038/s41467-020-18902-x (PMC7578651; doi:10.1038/s41467-020-18902-x)
Supplement: Supplementary file 1 — Supplementary Information [file 41467_2020_18902_MOESM1_ESM.pdf]

Supplementary Information

A fast impedance-based fast antimicrobial susceptibility test

Daniel C. Spencer[1], Tegan F. Paton[2], Timothy J.J. Inglis[2,3], J. Mark Sutton[4] and Hywel Morgan[1]

[1] Department of Electronics and Computer Science, and Institute for Life Science, University of Southampton, Hampshire, SO17 1BJ UK

[2] Department of Microbiology, PathWest Laboratory Medicine, WA, Nedlands, Australia

[3] Faculty of Health and Medical Sciences, University of Western Australia, Nedlands, Australia

[4] Public Health England, National Infection Service, Porton Down, Salisbury, Wiltshire, SP4 0JG, UK.

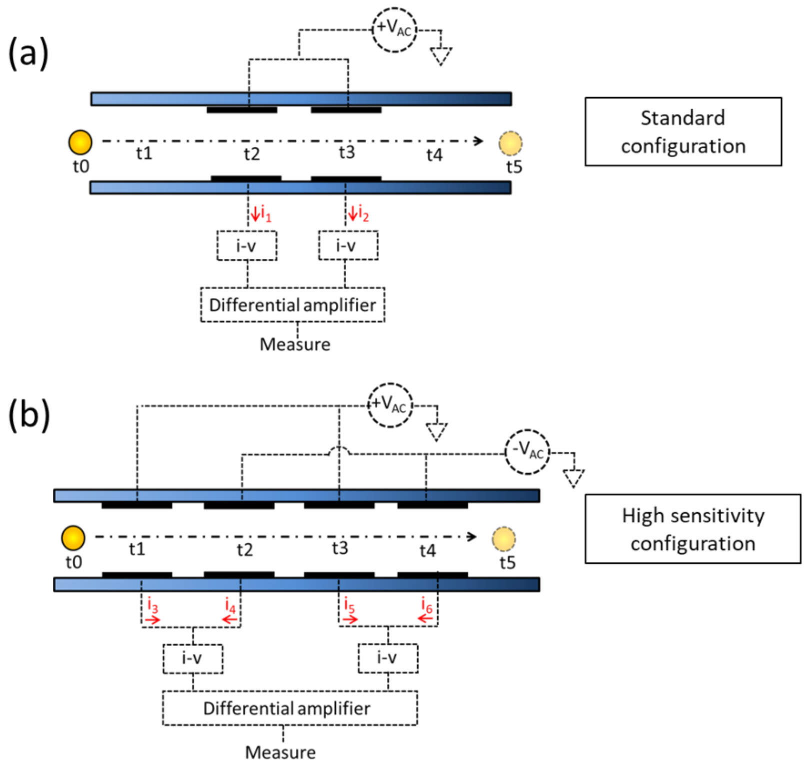

Supplementary Figure 1. Schematic of a conventional and new impedance cytometer electrode design. (a) shows the cross section of a conventional impedance cytometer, where cells suspended in an electrolyte flow along the channel one-by-one through two pairs of electrodes. An AC voltage at one of more frequencies is applied to the top two electrodes. The bottom two electrodes are each held at virtual ground through a transimpedance amplifier (i-v converter) and the difference between currents i1 and i2 is measured to determine the particle impedance, measured with a differential amplifier and lock-in amplifier. In (a) the steady-state currents i1 and i2 are relatively large, of the order of mA (suspending medium is a high salt buffer), whereas the change in the current produced by a passing particle of the size of a bacterial cell is in the range of nano-amps. To maximise the signal-to-noise ratio of the measurement signal, the applied voltage should be as high as possible and the gain in the current-to-voltage converters should be maximised. However, as the applied voltage is increased, i1 and i2 increase proportionally which leads to clipping in the i-v converters. To increase

sensitivity a new configuration that has four pairs of electrodes is used and is shown in **(b)**. Two of the top electrodes are driven with an AC voltage, whilst the other two top electrodes are driven with the same voltage signal, but phase shifted by 180°. Thus, currents i3 and i4 are out-of-phase by 180° and the sum of these is zero when there is no particle in the channel. This allows the drive voltage and gain in the current-voltage amplifiers to be increased substantially, enabling much smaller particles to be measure in a large dimension channel.

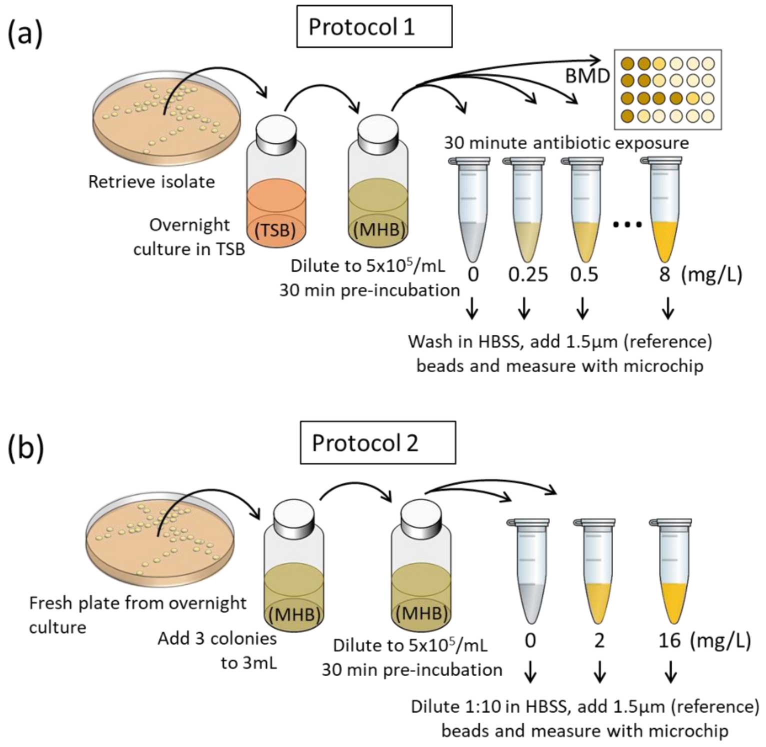

Supplementary Figure 2. Workflow for two different experimental protocols. (a) Experimental methodology for impedance-based minimum inhibitory concentration (MIC), protocol 1**.** A colony picked from a plate is incubated overnight in Tryptic Soy Broth (TSB) to the stationary phase. An aliquot of this culture is diluted into Mueller Hinton Broth (MHB) to a concentration of $5x10^5$ cells/mL and incubated at 37°C for 30 minutes to obtain an actively dividing culture. An aliquot of the actively dividing culture is used for a standard broth microdilution assay. Aliquots (950µL) are added to 7 pre-warmed test tubes each containing 50uL MHB and Meropenem to give a final antibiotic concentration of 0, 0.25, 0.5, 1, 2, 4 or 8 mg/L. The tubes are incubated for 30 minutes (antibiotic exposure). Cells are then washed once in hanks balanced salt solution (HBSS) and subsequently diluted 1:10 in HBSS. 1.5um diameter beads (reference particles) are added to each sample (10^4/mL). The sample is loaded into a syringe measured for 3 minutes at 30ul/min in the micro cytometer. **(b)** Experimental methodology for impedance based breakpoint analysis (protocol 2). Three colonies are selected from a plate and added to 3mL MHB. The sample is vortexed to re-suspend the bacteria and is then diluted to a concentration of $5x10^5$/mL in MHB. The sample is incubated for 30 minutes to

obtain an actively dividing culture. Aliquots of 500uL are added to test tubes containing pre-warmed 500uL MHB each with a final antibiotic concentration at the clinical breakpoint (2 or 16mg/L for Meropenem, 1 mg/L ciprofloxacin, 8 mg/L gentamicin, 4 mg/L Colistin, 8 mg/L ceftazidime, Amoxicillin/clavulanic acid and cefoxitin) along with a control (0 mg/L). Each tube is incubated for 30 minutes (antibiotic exposure), the sample is diluted 1:10 in HBSS, 1.5um diameter beads (reference particles) are added and the sample is measured for 2 minutes at 30ul/min in the micro cytometer.

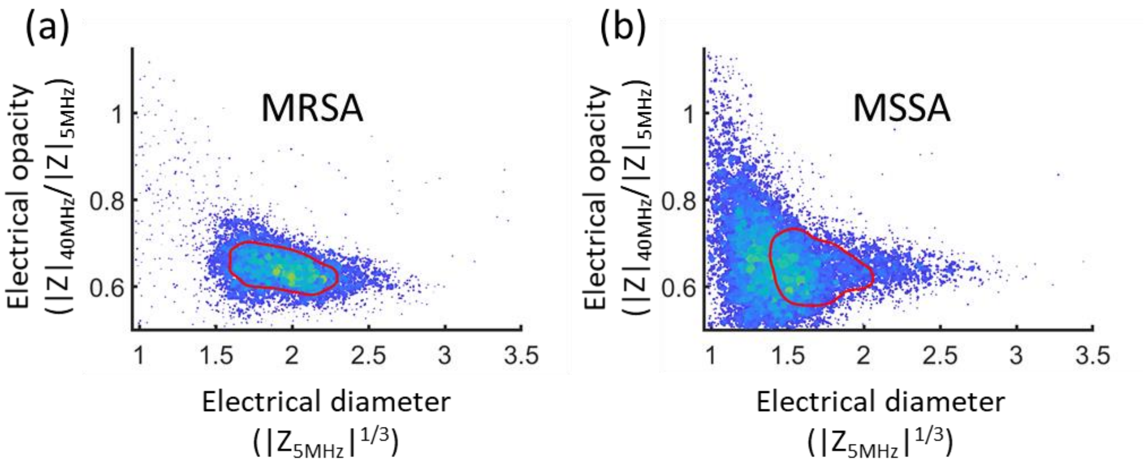

Supplementary Figure 3. **Scatterplots for methicillin sensitive and methicillin resistant *S. aureus* after exposure to cefoxitin.** The figure shows a scatterplot for (a) a methicillin resistant and (b) methicillin sensitive strain of *S. aureus* after a 30 minute exposure to cefoxitin which is used for testing methicillin resistance as per EUCAST guidelines. The *x*-axis is the cube root of the impedance at low frequency (proportional to diameter) measured at a frequency of 5MHz. The *y*-axis is the electrical opacity, a measure of membrane/cell wall properties normalised to cell volume, measured at 40MHz. The red contour defines the cell population before antibiotic exposure. Source data are provided as a Source Data file.

Figure 3

Species	Isolate Designation	Meropenem MIC by BMD (n=3)
Klebsiella pneumoniae	43358	<0.25 mg/L
Klebsiella pneumoniae	ATCC 700603	<0.25 mg/L
Klebsiella pneumoniae	18397	<0.25 mg/L
Klebsiella pneumoniae	43292	<0.25 mg/L
Klebsiella pneumoniae	44271	<0.25 mg/L
Klebsiella pneumoniae	KS1	8 mg/L
Klebsiella pneumoniae	KS11	8 mg/L
Klebsiella pneumoniae	K1	128 mg/L
Klebsiella pneumoniae	1705	128 mg/L
Klebsiella pneumoniae	K14	256 mg/L

Figure 4

Species	Isolate Designation	Meropenem Sensitivity
Acinetobacter baumannii	NCTC 13424	Resistant (OXA-23)
Acinetobacter baumannii	ATCC 17978	Sensitive
Klebsiella pneumoniae	51851	Resistant (KPC)
Klebsiella pneumoniae	NCTC 13368	Sensitive (SHV-18 ESBL)
Klebsiella pneumoniae	CFI-014	Carbapenemase negative, carbapenem resistant
Pseudomonas aeruginosa	NCTC 13437	Resistant (VIM-10)
Pseudomonas aeruginosa	PA01	Sensitive
Escherichia coli	CFL 161	Resistant (KPC-2)
Escherichia coli	NCTC 12923	Sensitive

Figure 5

Species	Isolate Designation	Antibiotic and Sensitivity
Klebsiella pneumoniae	51851	Colistin Resistant
Klebsiella pneumoniae	NCTC 13438	Colistin Sensitive
Klebsiella pneumoniae	51851	Ciprofloxacin Resistant
Klebsiella pneumoniae	TW3	Ciprofloxacin Sensitive
Klebsiella pneumoniae	NCTC 13368	Ceftazidime Resistant
Klebsiella pneumoniae	M6	Ceftazidime Sensitive
Klebsiella pneumoniae	NCTC 13368	Gentamicin Resistant
Klebsiella pneumoniae	NCTC 5054	Gentamicin Sensitive
Klebsiella pneumoniae	NCTC 13438	Co-amoxiclav Resistant
Klebsiella pneumoniae	M6	Co-amoxiclav Sensitive
Escherichia coli	LEC001	Ceftazidime Resistant
Escherichia coli	NCTC 12923	Ceftazidime Sensitive
Staphylococcus aureus	NCTC 13616	(MRSA) Cefoxitin Resistant (expresses mecA) MIC=32mg/L
Staphylococcus aureus	NCTC 6571	(MSSA) Cefoxitin Sensitive, MIC=1mg/L

Supplementary Table 1. **Bacterial isolate information for each experiment.** Please refer to the main text for more details for each experimental protocol.
