# Supplementary material for: A fast impedance-based antimicrobial susceptibility test
Source: Nat Commun. 2020 Oct 21;11:5328. doi: 10.1038/s41467-020-18902-x (PMC7578651; doi:10.1038/s41467-020-18902-x)
Supplement: Supplementary file 3 — Reporting Summary [file 41467_2020_18902_MOESM3_ESM.pdf]

nature research

Corresponding author(s): Prof Hywel Morgan

Last updated by author(s): Aug 12, 2020

Reporting Summary

Nature Research wishes to improve the reproducibility of the work that we publish. This form provides structure for consistency and transparency in reporting. For further information on Nature Research policies, see our Editorial Policies and the Editorial Policy Checklist.

Statistics

For all statistical analyses, confirm that the following items are present in the figure legend, table legend, main text, or Methods section.

n/a	Confirmed	
☐	☒	The exact sample size (n) for each experimental group/condition, given as a discrete number and unit of measurement
☐	☒	A statement on whether measurements were taken from distinct samples or whether the same sample was measured repeatedly
☐	☒	The statistical test(s) used AND whether they are one- or two-sided<br>*Only common tests should be described solely by name; describe more complex techniques in the Methods section.*
☒	☐	A description of all covariates tested
☒	☐	A description of any assumptions or corrections, such as tests of normality and adjustment for multiple comparisons
☐	☒	A full description of the statistical parameters including central tendency (e.g. means) or other basic estimates (e.g. regression coefficient) AND variation (e.g. standard deviation) or associated estimates of uncertainty (e.g. confidence intervals)
☐	☒	For null hypothesis testing, the test statistic (e.g. F, t, r) with confidence intervals, effect sizes, degrees of freedom and P value noted<br>*Give P values as exact values whenever suitable.*
☒	☐	For Bayesian analysis, information on the choice of priors and Markov chain Monte Carlo settings
☒	☐	For hierarchical and complex designs, identification of the appropriate level for tests and full reporting of outcomes
☒	☐	Estimates of effect sizes (e.g. Cohen's d, Pearson's r), indicating how they were calculated

Our web collection on statistics for biologists contains articles on many of the points above.

Software and code

Policy information about availability of computer code

Data collection	The impedance data was collected using ZiControl (version 19.05, Zurich Instruments, https://www.zhinst.com/)
Data analysis	All data analysis scripts were developed in MATLAB (version 2019a, Mathworks). Scripts are available on reasonable request. Statistical analysis performed using standard functions in MATLAB (version 2019a, Mathworks).

For manuscripts utilizing custom algorithms or software that are central to the research but not yet described in published literature, software must be made available to editors and reviewers. We strongly encourage code deposition in a community repository (e.g. GitHub). See the Nature Research guidelines for submitting code & software for further information.

Data

Policy information about availability of data

All manuscripts must include a data availability statement. This statement should provide the following information, where applicable:

- Accession codes, unique identifiers, or web links for publicly available datasets
- A list of figures that have associated raw data
- A description of any restrictions on data availability

All data supporting this study (Fig. 2 Fig. 3, Fig. 4, Fig. 5 and Supplementary Fig. 3) are openly available from the University of Southampton repository at https://doi.org/10.5258/SOTON/D1405

Field-specific reporting

Please select the one below that is the best fit for your research. If you are not sure, read the appropriate sections before making your selection.

- [x] Life sciences
- [ ] Behavioural & social sciences
- [ ] Ecological, evolutionary & environmental sciences

For a reference copy of the document with all sections, see nature.com/documents/nr-reporting-summary-flat.pdf

Life sciences study design

All studies must disclose on these points even when the disclosure is negative.

Sample size	No sample size calculations were performed. In general approximately 10,000 cells were measured per experiment which is in line with flow cytometry analysis of populations. This number of events per measurement condition are sufficient for robust determination of trends in the populations. Strain information is given in the Supplementary information table 1.
Data exclusions	As in conventional cytometry, coincident events were removed from the analysis.
Replication	All experiments were performed at least 3 times. We confirm all attempts at replicating the results were successful.
Randomization	Not relevant as experiments were carried out comparing the same bacterial isolate with and without exposure to a known antibiotic. This study focused on the introduction of a new technology and the electrical characterisation of the effect of antibiotic exposure to populations of single bacteria.
Blinding	Not relevant as this work was carried out on known bacterial isolates and the work focused on analysing the electrical changes with exposure to a known antibiotic.

Reporting for specific materials, systems and methods

We require information from authors about some types of materials, experimental systems and methods used in many studies. Here, indicate whether each material, system or method listed is relevant to your study. If you are not sure if a list item applies to your research, read the appropriate section before selecting a response.

Materials & experimental systems

n/a	Involved in the study	
☒	☐	Antibodies
☒	☐	Eukaryotic cell lines
☒	☐	Palaeontology and archaeology
☒	☐	Animals and other organisms
☒	☐	Human research participants
☒	☐	Clinical data
☒	☐	Dual use research of concern

Methods

n/a	Involved in the study	
☒	☐	ChIP-seq
☒	☐	Flow cytometry
☒	☐	MRI-based neuroimaging
